# A novel strategy for precise prognosis management and treatment option in colon adenocarcinoma with TP53 mutations

**DOI:** 10.3389/fsurg.2023.1079129

**Published:** 2023-02-09

**Authors:** Lei Niu, Langbiao Liu, Jun Cai

**Affiliations:** Department of General Surgery, Beijing Friendship Hospital, Capital Medical University, Beijing, China

**Keywords:** prognosis, molecular signature, colon cancer, TP53 mutant, precision treatment planning

## Abstract

**Background:**

TP53 is one of the most frequent mutated genes in colon cancer. Although colon cancer with TP53 mutations has a high risk of metastasis and worse prognosis generally, it showed high heterogeneity clinically.

**Methods:**

A total of 1,412 colon adenocarcinoma (COAD) samples were obtained from two RNA-seq cohorts and three microarray cohorts, including the TCGA-COAD (*N* = 408), the CPTAC-COAD (*N* = 106), GSE39582 (*N* = 541), GSE17536 (*N* = 171) and GSE41258 (*N* = 186). The LASSO-Cox method was used to establish the prognostic signature based on the expression data. The patients were divided into high-risk and low-risk groups based on the median risk score. The efficiency of the prognostic signature was validated in various cohorts, including TP53-mutant and TP53 wild-type. The exploration of potential therapeutic targets and agents was performed by using the expression data of TP53-mutant COAD cell lines obtained from the CCLE database and the corresponding drug sensitivity data obtained from the GDSC database.

**Results:**

A 16-gene prognostic signature was established in TP53-mutant COAD. The high-risk group had significantly inferior survival time compared to the low-risk group in all TP53-mutant datasets, while the prognostic signature failed to classify the prognosis of COAD with TP53 wild-type properly. Besides, the risk score was the independent poor factor for the prognosis in TP53-mutant COAD and the nomogram based on the risk score was also shown good predictive efficiency in TP53-mutant COAD. Moreover, we identified SGPP1, RHOQ, and PDGFRB as potential targets for TP53-mutant COAD, and illuminated that the high-risk patients might benefit from IGFR-3801, Staurosporine, and Sabutoclax.

**Conclusion:**

A novel prognostic signature with great efficiency was established especially for COAD patients with TP53 mutations. Besides, we identified novel therapeutic targets and potential sensitive agents for TP53-mutant COAD with high risk. Our findings provided not only a new strategy for prognosis management but also new clues for drug application and precision treatment in COAD with TP53 mutations.

## Introduction

Colon cancer is a type of cancer that begins in the large intestine which is the final part of the digestive tract. Colon adenocarcinoma (COAD) is the most common subtype of colon cancer. As reported by the world health organization (WHO), approximately 1.15 million new cases and 0.58 million new deaths of colon cancer in 2020, which ranks the fifth leading cause of cancer death around the world (1). Despite the disease burden of colon cancer have been decreased slightly but generally remaining stable in the last several years (2), China still had the largest new cases and death cases, accounting for 28.20% and 28.11% of the world's, respectively (3). Recently, many treatments have been developed to help inhibiting tumor progression, including surgery, radiation therapy, chemotherapy, targeted therapy, and immunotherapy (4–10). However, the outcomes of patients with colon cancer, especially of patients in the late stage, are still very poor and one of the most important reasons is that mutations of key driver genes promote the progression of colon cancer.

TP53 is a crucial tumor suppressor that responds to diverse stress signals by orchestrating specific cellular responses, including transient cell cycle arrest, cellular senescence and apoptosis, which are all processes associated with tumor suppression (11). However, inactivation of TP53 by mutations provides a selective advantage to tumor cells, enabling them to avoid cell cycle checkpoints, avoid apoptosis and senescence, promoting cancer cells proliferation abnormally (12), which enables TP53 mutations become one of the most important event for the tumorigenesis (13). TP53 mutations occur in over 50% types of cancer, which has become one of the most frequent alterations in human cancers. In colorectal cancer, approximately 60% of patients had TP53 mutations (14). Numerous studies have revealed that TP53 mutation was associated with the tumorigenesis and progression in colon cancer (15, 16), resulting in a poor prognosis. However, clinicians also found high heterogeneity in patients with TP53 mutant colon cancer. It is important to classify the TP53-mutant COAD with a more precise signature to help individual treatment. Recent study has developed a precise prognostic signature in patients with TP53-mutant hepatocellular carcinoma (17). However, the precise classification for prognosis management of patients with TP53-mutant COAD has not been developed.

In this study, we established a prognostic signature especially in COAD with TP53 mutations by bioinformatics analysis and evaluated it in both TP53-mutant COAD cohorts and TP53 wild-type cohorts. We also attempted to explore the potential targets and therapeutic agents for high-risk patients with TP53 mutant COAD, expecting to provide new insight to precision treatment for colon cancer with TP53 mutations.

## Methods

### RNA sequencing data collection

The transcriptome profile, mutation data and clinical follow-up information of 414 samples were downloaded from the Cancer Genome Atlas (TCGA) database (https://portal.gdc.cancer.gov). A prospectively collected colon cancer cohort from the Clinical Proteomic Tumor Analysis Consortium (CPTAC) was consistent of paired tumor and non-tumor colon tissues from 110 colon cancer patients (18). The RNA sequencing data, mutation data and clinical data were obtained from the cBioPortal database (https://www.cbioportal.org/study/summary?id=coad_cptac_2019). The two RNA-sequencing cohorts were merged into one combined RNA-seq cohort after removing the batch effect. A total of 261 TP53-mutant samples in the combined RNA-seq cohort were used for further analysis. The data we used was obtained from public database and the patients involved in the database have obtained ethical approval.

### Microarray data collection

A total of three microarray datasets were enrolled in this study. GSE39582 and GSE17536 were retrieved from the GPL570 platform, and GSE41258 were retrieved from the GPL96 platform. The expression data and corresponding clinical information of 585 samples in the GSE39582, 177 samples in the GSE17536 and 186 samples in the GSE41258 were downloaded from the Gene Expression Omnibus (GEO) database (https://www.ncbi.nlm.nih.gov/geo/). After eliminating samples without complete clinical data and survival data, 541 samples in the GSE39582, 177 samples in the GSE17536, and 182 samples in the GSE41258 were used for further analysis. Notably, 189 samples in the GSE39582 were recorded as TP53 mutate while other 352 samples were recorded as TP53 wild-type, and the TP53-mutant samples were used for signature establishment. GSE17536 and GSE41258 were combined into one GEO cohort as validation.

### Least absolute shrinkage and selection operator (LASSO) analysis

One hundred and eighty-nine samples with TP53 mutations in the GSE39582 were randomized into the training set (*N* = 94) and the testing set (*N* = 95). LASSO-Cox regression method (19, 20) was performed by the “glmnet” R package in the training set and the risk score of each sample were calculated based on the coefficient and expression level of each selected genes.

### Random forest (RF) analysis

To validate our result in a larger sample size resulting in achieving a more reliable finding, we performed RF analysis *via* the “RandomForest” R package to construct a TP53 mutation status prediction model in GSE39582. The TP53 mutation status of samples in the combined GEO cohort was estimated by the TP53 mutation status prediction model. Finally, one hundred and ninety-six samples in the combined GEO cohort were predicted as TP53 mutant samples.

### Cancer cell lines analysis

The Broad Institute Cancer Cell Line Encyclopedia (CCLE) project contains the expression profile data and somatic mutation data of over a thousand human cancer cell lines (21) and the data were downloaded from the CCLE website (https://portals.broadinstitute.org/ccle/). CERES score is a computational method to estimate gene dependency levels from CRISPR-Cas9 essentiality screens while accounting for the copy-number-specific effect (22). A more negative CERES score indicates that the gene is essential for cell viability in the certain cell line. The CERES scores of genome-scale CRISPR knockout screens for 18,333 genes in 739 cell lines were acquired from the dependency map (DepMap) portal (https://depmap.org/portal/).

### Drug sensitivity analysis

Genomics of Drug Sensitivity in Cancer (GDSC) is the largest public database that provides drug response data of over five hundred compounds in about one thousand human cancer cell lines (23). In the GDSC database, forty-nine colon and rectal adenocarcinomas (COAD-READ) cell lines were found and twenty-five of them harbor TP53 mutations. To analyze the drug response in TP53-mutant colon cancer, we downloaded the IC50 data of the drugs in the TP53-mutant COAD-READ cell lines in the GDSC database (https://www.cancerrxgene.org/). The information on the cell lines was downloaded from the DepMap portal (https://depmap.org/portal/).

### Statistical analysis

**Figure 1:**
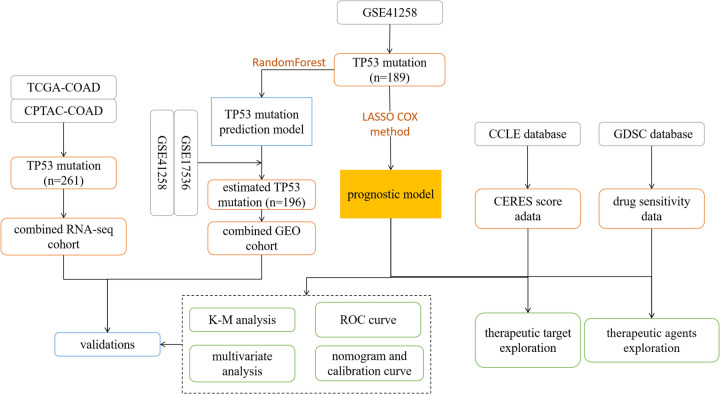
The flow chart for this study.

The survival curves were drawn by using Kaplan–Meier survival analysis *via* the “Survival” R package. The difference in survival time between the two groups was tested by a log-rank test. The time-dependent receiver operating characteristic (ROC) curve was performed *via* the “timeROC” R package, and the value of the area under the curve (AUC) was obtained as the criterion for the accuracy of the prognostic signature. Multivariate Cox analysis was used to evaluate the independence of our prognostic signature on the prognosis in TP53-mutant COAD. Nomogram and calibration curve were fabricated by “rms” R package. All the statistical analysis and visualization were performed by the R (version 4.0.2). A two-tail *p*-value < 0.05 was considered a significant difference. A flow chart of our study was shown in Figure 1.

## Results

### Establishment of a 16-gene prognostic signature in COAD with Tp53 mutations

**Figure 2:**
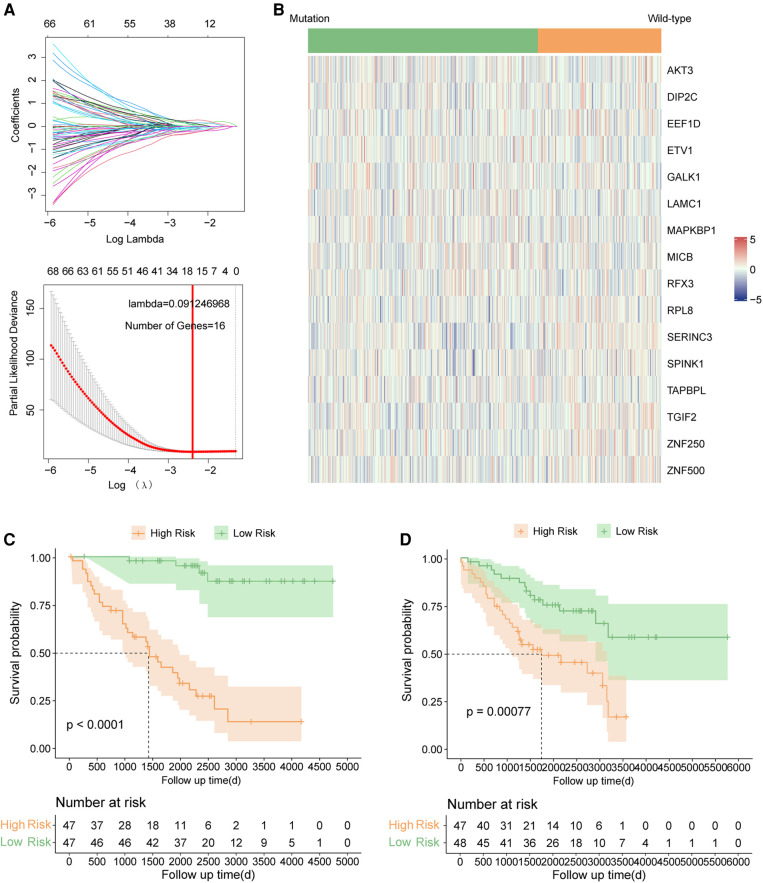
Signature construction in TP53 mutant COAD of GSE39582. (**A**) LASSO COX analysis. (**B**) Expression pattern of 16 signature genes. (**C**) Kaplan–Meier survival curve between high risk and low risk groups in the training set. (**D**) Kaplan–Meier survival curve between high risk and low risk groups in the testing set.

The clinical characteristics of all samples enrolled in this study were summarized in the Supplementary Table S1. Firstly, two hundred and fifty-three genes were identified as prognostic genes in TP53-mutant samples in the GSE39582 by univariate cox analysis (Supplementary Table S2, all *p* < 0.01). And then, TP53-mutant samples in the GSE39582 were randomized into the training set and the testing set. Based on the prognostic genes, we established a 16-gene prognostic signature by LASSO-Cox regression in the training set (Figure 2A). The selected 16 genes included GALK1, TGIF2, TAPBPL, SPINK1, ZNF500, LAMC1, MICB, RPL8, EEF1D, MAPKBP1, ZNF250, RFX3, ETV1, SERINC3, DIP2C, and AKT3. The risk score of each sample was calculated based on the expression level and the coefficient of each gene using the uniform formula: $\mathrm{risk}\;\mathrm{score}=\sum_{i=0}^{n}\exp\;r_{i}*\mathrm{coe}{\;f}_{i}$ (Supplementary Table S3). The expression profiles of these sixteen signature genes in GSE39582 were shown in Figure 2B.

### The 16-gene prognostic signature had great predictive efficiency in Tp53 mutant COAD

**Figure 3:**
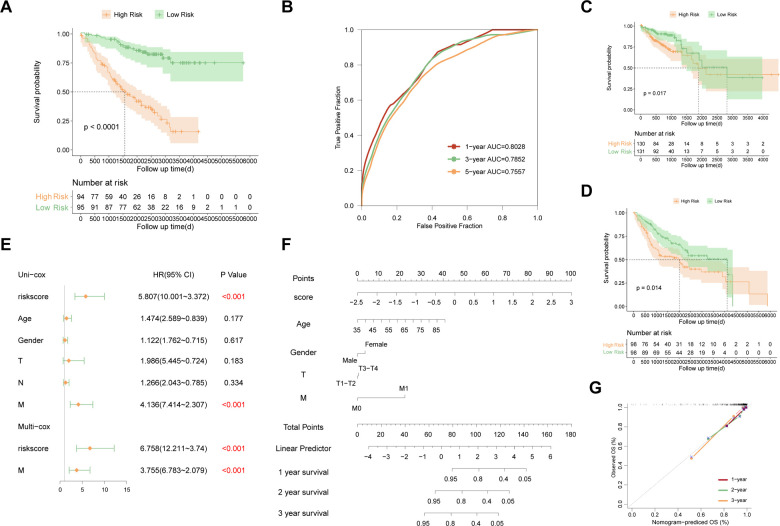
Evaluation and validation of the established signature in TP53 mutant COAD. (**A**) Kaplan–Meier survival curve between high risk and low risk groups in TP53 mutant samples of GSE39582. (**B**) Time-dependent ROC curve in TP53 mutant samples of GSE39582. (**C**) Kaplan–Meier survival curve between high risk and low risk groups in TP53 mutant samples of combined RNA-seq cohort. (**D**) Kaplan–Meier survival curve between high risk and low risk groups in estimated TP53 mutant samples of combined GEO cohort. (**E**) Multivariate analysis in TP53 mutant samples of GSE39582. (**F**) Nomogram based on the established signature in TP53 mutant samples of GSE39582. (**G**) Calibration curve of 1-year to 3-year survival predictive efficiency of the nomogram.

The risk scores of each sample were calculated by uniform formula, and the dataset was divided into high and low-risk groups based on the median value of risk scores. The clinical characteristics of each subgroup were summarized in Supplementary Table S4. Samples with high-risk scores had inferior outcomes compared to those with the low-risk score in the training set (Figure 2C, *p* < 0.0001), the testing set (Figure 2D, *p* = 0.00077), and the TP53 mutant samples in the GSE39582 cohort (Figure 3A, *p* < 0.0001). Besides, the AUC values of 1-year, 3-year, and 5-year survival prediction in TP53-mutant samples of GSE39582 were 0.8028, 0.7852, and 0.7557, respectively (Figure 3B). Moreover, the TP53-mutant samples in the combined RNA-seq cohort and the estimated TP53-mutant samples in the combined GEO cohort were enrolled as validation. Similarly, a high-risk score was associated with a worse prognosis in the TP53-mutant samples in the combined RNA-seq cohort (Figure 3C, *p* = 0.017) and the estimated TP53-mutant samples in the combined GEO cohort (Figure 3D, *p* = 0.014). In addition, the univariate-cox analysis showed that risk score and M category were prognostic factors in TP53-mutant COAD of GSE39582 (Figure 3E, all *p* < 0.001), thus we enrolled these two indexes for multivariate-cox analysis. Notably, risk score and M category were both independent poor factors for the prognosis in TP53-mutant COAD (Figure 3E, all *p* < 0.001). In the combined RNA-seq cohort, risk score, age, T category, N category, and M category were all poor factors for the prognosis in TP53-mutant COAD (Supplementary Figure S1A, all *p* < 0.05), besides, age and M stage were independent poor factors on the prognosis in TP53-mutant COAD (Supplementary Figure S1A, all *p* < 0.05). Finally, we fabricated the nomogram based on our signature and multiple clinical features for TP53 mutant patients with microarray data (Figure 3F) or RNA sequencing data (Supplementary Figure S1B). Besides, we also performed the calibration curve to test the predictive efficiency of the nomogram. The result indicated that both two nomograms had good efficiency in the prediction of 1-year to 3-year survival in TP53 mutant COAD patients (Figure 3G, Supplementary Figure S1C).

### The 16-gene prognostic signature failed to predict the prognosis in Tp53 wild-type COAD

**Figure 4:**
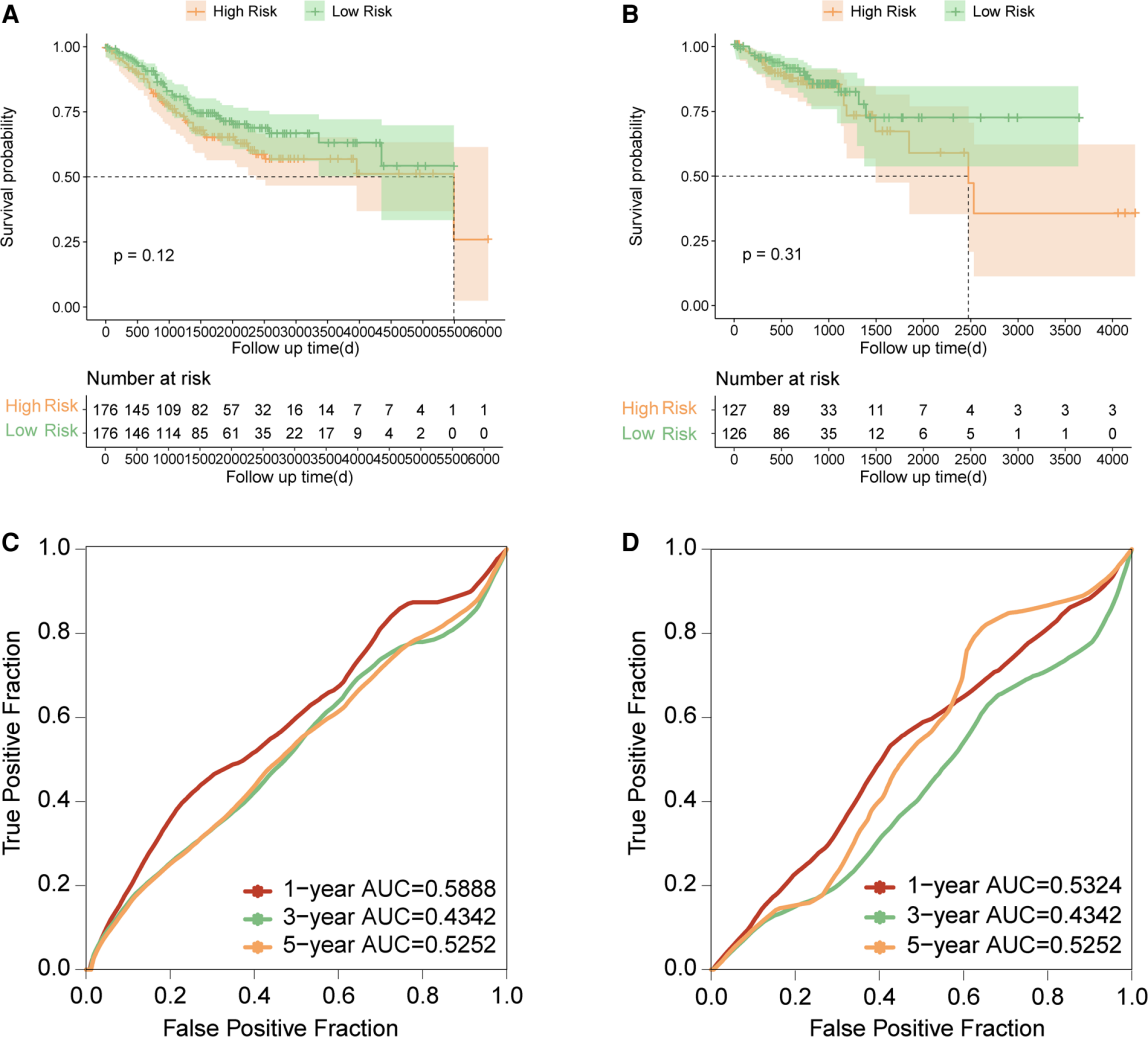
Evaluation and validation of the established signature in TP53 wild-type COAD. (**A**) Kaplan–Meier survival curve between high risk and low risk groups in TP53 wild-type samples of GSE39582. (**B**) Kaplan–Meier survival curve between high risk and low risk groups in TP53 wild-type samples of combined RNA-seq cohort. (**C**) Time-dependent ROC curve in TP53 wild-type samples of GSE39582. (**D**) Time-dependent ROC curve in TP53 wild-type samples of combined RNA-seq cohort.

To test whether the established signature can be used for prognosis prediction in COAD with TP53 wild-type, the prognostic significance of our signature were also evaluated in COAD samples with TP53 wild type, including 352 TP53 wild-type samples in the GSE39582 and 253 samples with TP53 wild-type in the combined RNA-seq cohort. The risk score of each sample were also calculated with uniform formula and each cohort were divided into high risk and low risk groups based on the median value of risk score, respectively. Comparison of survival curve between high risk and low risk groups showed that no significant difference was found in the survival between high risk group and low risk group in both the TP53 wild-type samples in the GSE39582 (Figure 4A, *p* = 0.12) and samples with TP53 wild-type in the combined RNA-seq cohort (Figure 4B, *p* = 0.31). Moreover, we estimated the predictive efficiency on the 1-year, 3-year, and 5-year survival of COAD with TP53 wild type in two cohorts using ROC curve analysis. The result both indicated that the established signature had poor predictive efficiency on the prognosis in TP53 wild-type COAD (Figures 4C,D). Taken together, the established signature failed to use for prognosis management in COAD with wild-type TP53, which indicated that our signature was especially used for prognosis prediction in TP53 mutant COAD patients.

### RHOQ, SGPP1 and PDGFRB might be the therapeutic targets for high risk patients

**Figure 5:**
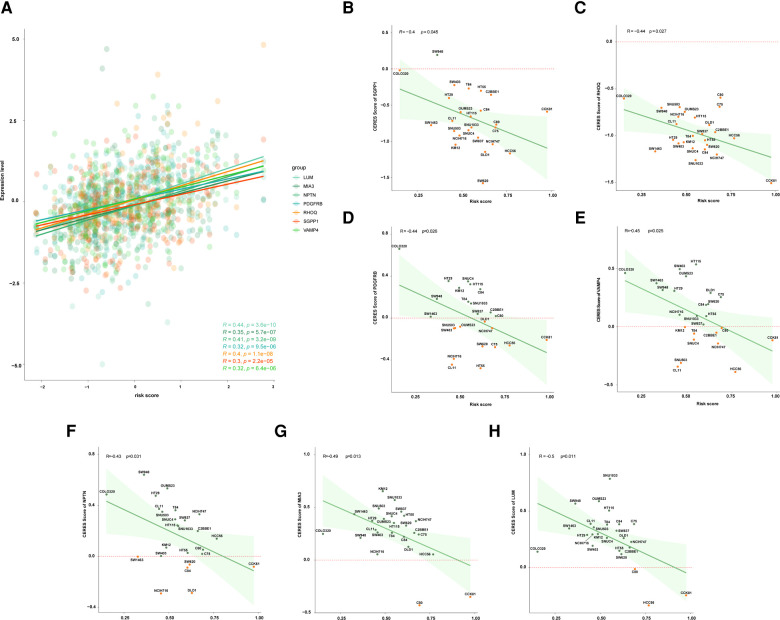
Exploration of potential therapeutic target for patients with high risk. (**A**) The correlation between risk score and expression level of LUM, MIA3, NPTN, PDGFRB, RHOQ, SGPP1, and VAMP4. (**B**) The correlation between risk score and the CERES score of SGPP1 in multiple TP53 mutant COAD cell lines. (**C**) The correlation between risk score and the CERES score of RHOQ in multiple TP53 mutant COAD cell lines. (**D**) The correlation between risk score and the CERES score of PDGFRB in multiple TP53 mutant COAD cell lines. (**E**) The correlation between risk score and the CERES score of VAMP4 in multiple TP53 mutant COAD cell lines. (**F**) The correlation between risk score and the CERES score of NPTN in multiple TP53 mutant COAD cell lines. (**G**) The correlation between risk score and the CERES score of MIA3 in multiple TP53 mutant COAD cell lines. (**H**) The correlation between risk score and the CERES score of LUM in multiple TP53 mutant COAD cell lines.

The expression data of 49 COAD cell lines were obtained from the CCLE database. Firstly, the correlation between the expression level of genes and risk score was performed, and the expression level of seven genes was positively correlated with a risk score, including LUM, MIA3, NPTN, PDGFRB, PHOQ, SGPP1, and VAMP4 (Figure 5A). And then, we found that 15 of the 49 COAD cell lines harbored TP53 mutations, thus, we further analyzed the relationship between the CERES score of seven above-mentioned genes and the risk score in the 15 TP53-mutant COAD cell lines. The result showed that the risk score was negatively correlated with the CERES score of all seven genes (Figures 5B–H). Finally, we defined three of the seven genes as an essential target for high-risk TP53-mutant COAD, which with CERES scores less than zero in 80% TP53 mutant COAD cell lines, including SGPP1, RHOQ, and PDGFRB.

### Exploration of potential therapeutic agents for Tp53-mutant COAD with high risk

**Figure 6:**
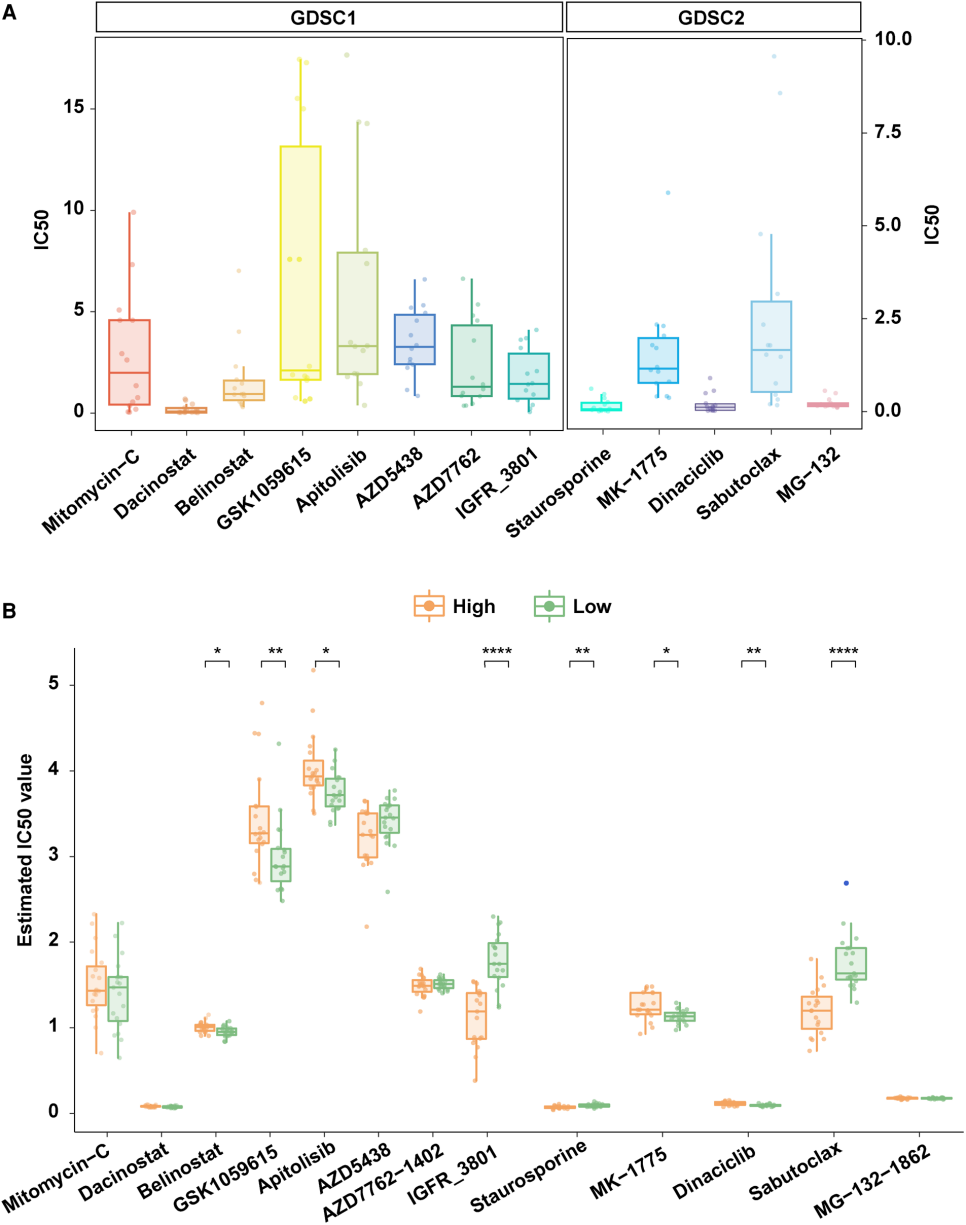
Exploration of potential therapeutic agents for patients with high risk. (**A**) TP53 mutant COAD cell lines were sensitive to multiple anti-tumor agents in the GDSC database. (**B**) Comparison of the IC50 of anti-tumor agents that were TP53 mutant COAD cell lines sensitive in high risk and low risk patients.

The sensitivity data of 518 anti-tumor drugs was obtained from the GDSC database which contained two datasets (GDSC1 and GDSC2). We identified candidate drugs by analyzing the IC50 of drugs in both two datasets. We found that the 15 TP53-mutant COAD cell lines were sensitive to eight drugs in the GDSC1 dataset and five drugs in the GDSC2 dataset, including Mitomycin-C, Dacinostat, Belinostat, GSK1059615, Apitolisib, AZD5438, AZD7762, IGFR-3801, Staurosporine, MK-1775, Dinaciclib, Sabutoclax, and MG-132 (Figure 6A). Moreover, we compared the IC50 between samples with high risk and low risk, and the result showed that the high-risk group had lower IC50 compared to the low-risk group in three drugs including IGFR-3801, Staurosporine, and Sabutoclax (Figure 6B), which indicated that IGFR-3801, Staurosporine, and Sabutoclax were potential agents for high-risk COAD with TP53 mutations.

## Discussion

TP53 is one of the most important driver genes for tumorigenesis and mutated or silenced in most human cancers including COAD (24). Top to 60% of patients with COAD harbors TP53 mutations, however, the prognostic value of TP53 mutation status in COAD is controversial (25, 26). Therefore, developing a more precise risk classification is helpful for prognostic management and precision treatment of patients with TP53 mutant COAD. In this study, we established a prognostic signature particular for COAD patients with TP53 mutations by 16-genes including GALK1, TGIF2, TAPBPL, SPINK1, ZNF500, LAMC1, MICB, RPL8, EEF1D, MAPKBP1, ZNF250, RFX3, ETV1, SERINC3, DIP2C, and AKT3. Among these sixteen genes, MICB have been reported as a biomarker for good prognosis in colorectal cancer (27). Besides, AKT3 enhanced migration and induce drug resistance in colon cancer cells by mediating miR-125b-5p (28). Interestingly, the established prognostic signature can distinguish COAD with worse outcome as high risk only in those with TP53 mutations instead of in those with TP53 wild-type, which suggested that the established prognostic signature had potential to be a novel risk classification especially for patients with TP53-mutant COAD. Moreover, the nomogram with great predictive efficiency on 1-year, 3-year, and 5-year survival in COAD with TP53 mutations was fabricated to improve the clinical application of our prognostic signature.

Nowadays, some prospective studies have reported that VEGF/VEGFR inhibitors and WEE1 inhibitors were promising therapies for patients with TP53 mutations (29, 30), however, the efficiency of these inhibitors in COAD with TP53 mutations has not been investigated yet. Therefore, the treatment of COAD patients with TP53 mutations is still a very tricky task for clinicians. It is urgent to the identification of novel drug targets for therapy development in TP53-mutant COAD resulting in improving patients' outcomes. In this study, we further classified the TP53-mutant COAD into high-risk and low-risk. Low-risk patients had good prognosis, suggesting that these patients might benefit from traditional treatment. However, the treatment decision for high-risk patients with poor prognosis needs to be very careful. Therefore, we explored the potential therapeutic targets for high-risk patients, expecting to help precision treatment, including SGPP1, RHOQ, and PDGFRB. SGPP1 is a catalyze of Sphingosine-1-phosphate (S1P), the latter is a bioactive sphingolipid metabolite that regulates diverse biologic processes (31, 32), and SIP is linked to signal transducer and activator of transcription 3 (STAT3) activation and the development of colitis-associated colorectal cancer (32). Some studies have demonstrated that SGPP1 was the target in the carcinogenesis and progression of colorectal cancer (33, 34). RHOQ is a member of the small Rho GTPase family, which cycles between an inactive GDP and an active GTP bound state (35). A study has illuminated that dysregulation of RNA editing in RHOQ contributed to the progression of colorectal cancer (36).

The development of a new drug is time-consuming. However, exploration of the new application of existing drugs can greatly shorten the time, which enables patients to benefit from treatment as soon as possible. Therefore, we further explored the potential sensitive drugs for high-risk patients, including IGFR-3801, Staurosporine, and Sabutoclax, expecting to provide new clue for clinical trial. IGFR-3801 is a drug targeting IGFR1 and the IGF1R signaling pathway. A study has demonstrated that the expression level of IGF1R within the nuclear compartment might lead to metastasis and therapies resistance in colorectal cancer (37). Staurosporine is a broad spectrum kinase inhibitor targeting to RTK signaling pathway. A study also demonstrated that Staurosporine inhibited cell growth by targeting the Hippo pathway (38). Multiple studies have demonstrated the important function of Staurosporine in colon cancer, including apoptosis-inducing, cisplatin chemoresistance alleviating, and carcinoembryonic antigen expression increasing (39–41). Sabutoclax is a small-molecule BH3 mimetic that inhibits the function of antiapoptotic Bcl-2 proteins and targets to apoptosis regulation pathway. A study has revealed that a combination of Sabutoclax and a serotype chimeric adenovirus improved therapeutic efficacy in colorectal cancer cells through enhancing the delivery of mda-7/IL-24 (42).

In conclusion, we established a more precise prognostic signature for TP53-mutant COAD, which might be useful for prognosis monitoring and treatment planning of patients with TP53 mutant COAD clinically. Besides, we further identified three potential therapeutic targets and three potential therapeutic agents for high-risk patients, expecting to help precision therapy development for TP53-mutant COAD.

Even so, there are still some limitations in this study. We constructed a TP53 mutation status prediction model by machine learning method to achieve more reliable results. However, there is still a slight difference between the estimated TP53 mutation status and the actual TP53 mutation status. Additionally, we explored the potential therapeutic targets and agents for TP53-mutant patients, but the effectiveness of these targets and agents in COAD with TP53 mutations still needs to be confirmed by further experiments. In the future, we will attempt to overcome these shortcomings.

## Conclusion

A 16-gene prognostic signature was established especially for COAD patients with TP53 mutations, which had great performance on the prognosis prediction in TP53-mutant COAD. Besides, we identified potential therapeutic targets and drugs for high-risk TP53-mutant COAD. Our findings provided not only a new strategy for prognosis management but also new clues for drug application and precision treatment in COAD with TP53 mutations.

## Data Availability

Publicly available datasets were analyzed in this study. This data can be found here: TCGA-COAD dataset: https://portal.gdc.cancer.gov; CPTAC-COAD dataset: https://www.cbioportal.org/study/summary?id=coad_cptac_2019; GSE39582, GSE17536, GSE41258: https://www.ncbi.nlm.nih.gov/geo/; CCLE database: https://portals.broadinstitute.org/ccle/; GDSC database: https://www.cancerrxgene.org/.
