# Supplementary material for: A novel strategy for precise prognosis management and treatment option in colon adenocarcinoma with TP53 mutations
Source: Front Surg. 2023 Feb 9;10:1079129. doi: 10.3389/fsurg.2023.1079129 (PMC9947352; doi:10.3389/fsurg.2023.1079129)
Supplement: Supplementary file 1 [file Datasheet1.pdf]

Supplementary Table 1. Clinical characteristics of all samples enrolled in this study

characterstics	Overall/n(%)	TCGA-COAD/n(%)	CPTAC-COAD	GSE39582/n(%)	GSE17536/n(%)	GSE41258/n(%)
n	1412	408	106	541	171	186
Age	437/974 (31.0/69.0)					
<60 years	437(31.0)	127(31.1)	38(35.8)	151(28.0)	56(32.7)	65(34.9)
≥60 years	974(69.0)	281(68.9)	68(64.2)	389(72.0)	115(67.3)	121(65.1)
Gender	663/749 (47.0/53.0)					
Female	663(47.0)	189(46.3)	63(59.4)	244(45.1)	78(45.6)	89(47.8)
Male	749(53.0)	219(53.7)	43(40.6)	297(54.9)	93(54.4)	97(52.2)
T						
T0	2 (0.2)	1 (0.2)	0 (0.0)	1 (0.2)	NA	0 (0.0)
T1	26 (2.1)	11 (2.7)	0 (0.0)	11 (2.1)	NA	4 (2.2)
T2	165 (13.5)	71 (17.4)	16 (15.1)	43 (8.3)	NA	35 (18.8)
T3	841 (68.9)	281 (68.9)	76 (71.7)	351 (67.4)	NA	133 (71.5)
T4	184 (15.1)	44 (10.8)	14 (13.2)	112 (21.5)	NA	14 (7.5)
Tis	3 (0.2)	0 (0.0)	0 (0.0)	3 (0.6)	NA	0 (0.0)
N						
N0	689 (56.4)	243 (59.6)	56 (52.8)	297 (57.0)	NA	93 (50.0)
N1	300 (24.6)	93 (22.8)	34 (32.1)	124 (23.8)	NA	49 (26.3)
N2	226 (18.5)	72 (17.6)	16 (15.1)	94 (18.0)	NA	44 (23.7)
N3	6 (0.5)	0 (0.0)	0 (0.0)	6 (1.2)	NA	0 (0.0)
M						
M0	890 (80.3)	303 (75.6)	NA	458 (87.9)	NA	129 (69.4)
M1	171 (15.4)	53 (13.2)	NA	61 (11.7)	NA	57 (30.6)
MX	47 (4.2)	45 (11.2)	NA	2 (0.4)	NA	0 (0.0)
Stage						
Stage I-II	213(15.2)	159(40.1)	54(50.9)	0(0.0)	0(0.0)	0(0.0)
Stage III-IV	1188(84.8)	238(59.9)	52(49.1)	541(100.0)	171(100.0)	186(100.0)

NA: Not applicable.

Supplementary Table 2. Prognostic genes in COAD with TP53 mutation

Gene	HR	upper95	lower95	p value
ABCD4	2	3.154	1.263	0.00263
ACACA	0.553	0.871	0.351	0.00945
ACOT8	0.533	0.84	0.338	0.00585
ACSF2	0.498	0.788	0.315	0.00239
ACTR3B	0.537	0.849	0.34	0.00674
ACTR5	0.501	0.792	0.317	0.00255
ADAM9	1.99	3.149	1.262	0.00253
ADRM1	0.4	0.641	0.249	8.23E-05
AGK	2.04	3.231	1.285	0.00199
AHCY	0.429	0.684	0.269	0.000249
AKAP1	0.534	0.844	0.338	0.00633
AKT3	2.02	3.192	1.273	0.00226
AP4S1	1.83	2.885	1.162	0.00831
ARFRP1	0.422	0.671	0.265	0.000171
ARRB1	0.467	0.742	0.294	0.000971
ASXL1	0.506	0.8	0.32	0.00296
ATP5H	2.23	3.536	1.407	0.00046
AUTS2	0.53	0.836	0.335	0.00562
BCS1L	0.539	0.851	0.341	0.00696
C19orf24	0.481	0.761	0.303	0.00141
C3orf52	1.86	2.935	1.177	0.00687
C8orf33	0.446	0.71	0.28	0.000467
CCDC28A	1.82	2.875	1.153	0.00921
CCDC28B	0.543	0.856	0.345	0.00757
CCDC94	0.388	0.623	0.242	4.67E-05
CCPG1	2.01	3.188	1.263	0.00258
CCR8	1.84	2.912	1.162	0.00841
CD2	0.506	0.799	0.321	0.00294
CDCA3	0.511	0.81	0.323	0.00367
CDK5RAP1	0.532	0.837	0.337	0.00554
CEBPA	0.458	0.728	0.288	0.000718
CEL	0.526	0.831	0.333	0.00502
CELP	0.481	0.762	0.304	0.00144
CKMT2	0.374	0.598	0.234	1.99E-05
CNNM3	0.47	0.747	0.295	0.00109
COL16A1	1.83	2.875	1.165	0.00772
COL1A1	1.81	2.857	1.152	0.00894
COL3A1	1.82	2.867	1.16	0.00808
COLEC12	1.88	2.961	1.193	0.00565
CSNK1G2	0.553	0.873	0.35	0.00971
CTNNBL1	0.379	0.608	0.236	2.86E-05

CXCR3	0.497	0.785	0.315	0.00221
CYP27A1	0.525	0.83	0.333	0.00504
DBI	0.501	0.794	0.317	0.0027
DCAKD	0.526	0.835	0.331	0.00554
DCTPP1	0.514	0.814	0.325	0.00393
DCUN1D4	1.99	3.159	1.249	0.00309
DDX23	0.534	0.845	0.337	0.00653
DDX27	0.502	0.792	0.318	0.00259
DDX49	0.495	0.784	0.312	0.00217
DFFB	0.489	0.778	0.308	0.00204
DIP2C	1.86	2.927	1.181	0.00648
DKK3	1.86	2.924	1.178	0.00674
DNAJA4	2.17	3.448	1.365	0.000773
DPM2	0.547	0.87	0.344	0.00964
DRG1	0.484	0.766	0.305	0.00156
DTX4	0.533	0.844	0.337	0.00632
DYNLRB1	0.486	0.77	0.307	0.00166
DYSF	1.82	2.856	1.156	0.00851
EDEM2	0.412	0.659	0.258	0.000131
EDEM3	2.05	3.239	1.298	0.00167
EEF1D	0.521	0.826	0.329	0.00473
EFNB2	1.91	3.014	1.208	0.00477
EIF2B1	0.544	0.858	0.345	0.00793
EIF4E2	0.533	0.846	0.335	0.00665
EIF4G3	1.86	2.922	1.179	0.00657
EIF6	0.516	0.818	0.326	0.00421
EMG1	0.518	0.818	0.328	0.00413
EPB41L1	0.448	0.71	0.282	0.00045
ERAP2	1.84	2.9	1.17	0.00751
ESM1	2.03	3.212	1.286	0.00194
ETV1	1.95	3.071	1.235	0.00351
EXOSC4	0.542	0.855	0.343	0.00754
FAM135A	1.85	2.92	1.174	0.00706
FARSA	0.501	0.793	0.316	0.00256
FBL	0.463	0.736	0.291	0.000822
FGF2	1.99	3.144	1.254	0.00283
FKBP10	2.17	3.443	1.364	0.000791
G0S2	1.94	3.066	1.227	0.00397
G3BP2	1.91	3.026	1.212	0.00453
GALK1	0.473	0.753	0.298	0.00123
GCDH	0.535	0.845	0.339	0.00645
GEMIN7	0.514	0.814	0.325	0.00382
GRB7	0.526	0.831	0.333	0.005
GTPBP8	0.545	0.858	0.346	0.00788

GZMB	0.542	0.852	0.344	0.00714
H2AFJ	0.474	0.753	0.298	0.00125
HCFC2	2.08	3.308	1.31	0.00147
HN1L	0.468	0.747	0.293	0.00113
HNRNPD	0.503	0.796	0.317	0.00275
HNRNPM	1.85	2.927	1.173	0.00711
HOXB2	2.19	3.472	1.383	0.000621
HSD17B10	0.531	0.838	0.336	0.00558
HSD17B8	0.526	0.833	0.332	0.00521
HSPA13	1.82	2.872	1.155	0.00873
HUNK	0.537	0.846	0.341	0.00674
HUWE1	1.89	2.989	1.197	0.00537
IFI35	0.524	0.829	0.331	0.00496
IL12RB1	0.521	0.822	0.331	0.00438
IL22RA1	0.492	0.778	0.311	0.00198
INO80B	0.5	0.792	0.316	0.00257
IRF2BP1	0.43	0.686	0.269	0.000263
ITGA1	1.81	2.855	1.15	0.00909
ITGBL1	2.2	3.5	1.381	0.000657
KIAA0753	1.81	2.851	1.151	0.00919
KIF3B	0.531	0.836	0.337	0.00548
KRT23	0.472	0.747	0.298	0.00105
LAMC1	1.91	3.002	1.214	0.0044
LGR5	0.53	0.837	0.335	0.00558
LHFPL2	1.84	2.901	1.163	0.00788
LIG1	0.55	0.869	0.348	0.00919
LIMCH1	1.93	3.047	1.224	0.00389
LSG1	0.522	0.826	0.331	0.00475
LSM14A	0.519	0.823	0.328	0.00464
LSM6	0.502	0.796	0.317	0.00272
LSM7	0.411	0.657	0.258	0.000121
LUM	1.95	3.075	1.233	0.00359
LYVE1	1.88	2.974	1.187	0.00621
MAN1A1	2.02	3.198	1.275	0.00217
MAPK14	0.515	0.813	0.326	0.00375
MAPKBP1	1.94	3.058	1.23	0.00382
MAT2A	0.532	0.84	0.337	0.00605
MCM2	0.527	0.835	0.333	0.00555
MCRS1	0.513	0.813	0.324	0.00381
MICB	0.535	0.848	0.338	0.00687
MLLT11	1.9	2.992	1.203	0.00509
MOCS3	0.495	0.784	0.313	0.00223
NAB1	2.04	3.224	1.291	0.00181
NAGPA	0.54	0.853	0.342	0.00734

NARF	0.483	0.769	0.304	0.00164
NAT6	0.533	0.842	0.338	0.00596
NDEL1	2.02	3.193	1.277	0.00218
NDUFB11	0.492	0.779	0.31	0.00196
NDUFB7	0.484	0.771	0.303	0.00177
NFS1	0.539	0.849	0.342	0.00684
NLE1	0.541	0.855	0.343	0.0074
NMT2	0.554	0.873	0.352	0.00998
NOTCH2	1.81	2.842	1.148	0.00953
NUP93	0.44	0.703	0.275	0.000405
OGFR	0.534	0.846	0.337	0.00651
OSBPL2	0.51	0.806	0.323	0.00327
PAF1	0.526	0.836	0.331	0.00558
PCIF1	0.378	0.606	0.236	2.64E-05
PDE1A	1.96	3.101	1.241	0.00319
PIK3CG	1.92	3.034	1.217	0.0045
PIPOX	0.47	0.745	0.296	0.000989
PLAGL2	0.486	0.77	0.307	0.0017
PLK2	1.82	2.862	1.154	0.00884
PLN	1.81	2.846	1.148	0.00946
PMM1	0.503	0.799	0.317	0.00301
PNN	1.81	2.851	1.148	0.00949
POLR2C	0.442	0.708	0.276	0.00049
POMT1	0.551	0.871	0.349	0.00993
POP1	0.551	0.865	0.35	0.00881
POP4	0.547	0.864	0.347	0.0087
PPDPF	0.543	0.86	0.343	0.0082
PPIC	1.81	2.842	1.149	0.00918
PRKAR1A	1.82	2.882	1.155	0.00895
PRKCZ	0.528	0.836	0.333	0.00572
PRMT7	0.436	0.699	0.272	0.000372
PRR15L	0.534	0.846	0.337	0.00652
PSMA7	0.518	0.817	0.329	0.00397
PSMB3	0.51	0.81	0.321	0.00367
PSMB7	0.539	0.85	0.341	0.0071
PSMD8	0.544	0.861	0.343	0.00833
PSMG1	0.494	0.781	0.313	0.00206
PTEN	1.86	2.945	1.172	0.00741
PTPN1	0.513	0.811	0.325	0.00359
PUF60	0.543	0.861	0.343	0.0084
PXMP4	0.435	0.694	0.273	0.000332
PYCRL	0.506	0.802	0.319	0.00312
QDPR	0.553	0.871	0.352	0.00947
QSER1	1.82	2.863	1.154	0.00878

RAB23	1.91	3.028	1.207	0.00483
RAE1	0.493	0.782	0.311	0.00213
RALY	0.448	0.714	0.28	0.000535
RBM42	0.549	0.866	0.348	0.00901
RECQL4	0.531	0.838	0.336	0.00579
RFX3	1.85	2.925	1.173	0.00726
RFX7	1.94	3.066	1.229	0.00376
RFXANK	0.468	0.746	0.293	0.00107
RGS2	2	3.155	1.265	0.00249
RNF114	0.344	0.554	0.213	4.35E-06
RPL8	0.529	0.836	0.335	0.00551
RPS21	0.458	0.725	0.29	0.000627
RPS25	1.89	2.995	1.194	0.00583
SDC4	0.401	0.64	0.251	7.50E-05
SDS	2.04	3.236	1.286	0.00199
SEC11A	2.25	3.583	1.418	0.000419
SEC24D	1.82	2.862	1.162	0.00794
SEMA5A	0.522	0.827	0.33	0.00482
SERINC3	0.505	0.797	0.32	0.00286
SGCB	1.94	3.067	1.223	0.00405
SIRPG	0.476	0.754	0.3	0.00121
SLC17A9	0.499	0.792	0.314	0.00261
SLC37A4	0.493	0.782	0.311	0.00212
SLC48A1	0.532	0.84	0.337	0.00591
SLC5A6	0.523	0.825	0.332	0.00447
SLC9A8	0.476	0.756	0.299	0.00131
SMARCAL1	0.451	0.718	0.284	0.000549
SND1	0.535	0.848	0.338	0.00652
SOD3	0.547	0.866	0.345	0.00897
SPATA2	0.474	0.752	0.299	0.00116
SPINK1	0.526	0.83	0.333	0.00501
SPP1	1.91	3.019	1.204	0.00511
STARD7	0.486	0.767	0.307	0.00155
STEAP4	1.85	2.929	1.175	0.00724
STRN3	2.01	3.2	1.267	0.0025
STX7	1.88	2.971	1.191	0.00584
TAF5L	0.537	0.847	0.341	0.00661
TAOK2	0.538	0.855	0.338	0.00764
TAPBPL	0.456	0.726	0.287	0.00068
TBC1D19	1.91	3.013	1.212	0.00457
TCFL5	0.535	0.841	0.34	0.00596
TELO2	0.51	0.811	0.321	0.00377
TGIF2	0.446	0.708	0.28	0.000448
THBS2	1.84	2.913	1.168	0.00756

TIMM13	0.422	0.674	0.265	0.000195
TMEM160	0.545	0.864	0.345	0.00862
TMEM45A	1.81	2.845	1.147	0.00962
TPP2	0.55	0.868	0.348	0.00907
TPX2	0.494	0.781	0.313	0.00209
TRAFD1	0.483	0.765	0.305	0.00153
TREM1	1.83	2.878	1.158	0.00853
TRIAP1	0.535	0.842	0.34	0.0058
TRIM31	0.511	0.812	0.322	0.00376
TROAP	0.538	0.85	0.341	0.0069
TRPC4AP	0.546	0.862	0.345	0.00852
TSEN34	0.518	0.82	0.327	0.00428
TTPAL	0.518	0.818	0.328	0.00412
TUBA1C	0.481	0.762	0.303	0.00146
UBA6	1.95	3.078	1.235	0.00345
UBE2C	0.496	0.782	0.315	0.00211
ULBP2	1.87	2.949	1.188	0.00615
USE1	0.544	0.859	0.344	0.00788
VIM	2.33	3.718	1.46	0.000255
WDR18	0.515	0.818	0.324	0.0042
WDR26	2.21	3.497	1.392	0.000556
WTAP	2.34	3.739	1.463	0.000248
XIAP	1.87	2.944	1.186	0.00609
YTHDF1	0.541	0.852	0.343	0.00716
YWHAB	0.506	0.797	0.322	0.00271
ZC3H4	0.472	0.747	0.298	0.00104
ZC4H2	0.505	0.8	0.319	0.00295
ZFP64	0.454	0.721	0.285	0.000618
ZNF16	0.439	0.699	0.276	0.000366
ZNF212	0.479	0.761	0.301	0.00144
ZNF250	0.55	0.866	0.349	0.00897
ZNF500	0.541	0.855	0.343	0.0075
ZNF552	0.494	0.786	0.311	0.00236
ZNF576	0.504	0.801	0.317	0.003
ZNF589	0.551	0.87	0.349	0.00946
ZNF839	1.85	2.915	1.17	0.00746

Supplementary Table 3.coefficient of genes in the prognostic signature

Gene	coefficient
AKT3	0.118293659
DIP2C	0.153988932
EEF1D	-0.017617322
ETV1	0.089835179
GALK1	-0.317510946
LAMC1	0.179853497
MAPKBP1	0.211583393
MICB	-0.102007514
RFX3	0.151787915
RPL8	-0.044596324
SERINC3	-0.141400266
SPINK1	-0.129678785
TAPBPL	-0.106307708
TGIF2	-0.211995834
ZNF250	-0.039743326
ZNF500	-0.111274939

Supplementary Table 4. Clinical feature between high risk and low risk groups in the GSE39582, combined GEO cohort and the combined RNA-seq cohort

Charateristics	GSE39582				combined GEO cohort				combined RNA-seq cohort			
	Overall/n(%)	Low risk group/n(%	High risk group/n(%	p-value#	Overall/n(%)	Low risk group/n(%	High risk group/n(%	p-value#	Overall/n(%)	Low risk group/n(%	High risk group/n(%	p-value#
n	189	94	95		196	98	98		263	131	132	
Age				1				0.278				0.628
<60 years	48(25.4)	24(25.5)	24(25.3)		60(30.6)	26(26.5)	34(34.7)		85(32.3)	40(30.5)	45(34.1)	
≥60 years	141(74.6)	70(74.5)	71(74.7)		136(69.4)	72(73.5)	64(65.3)		178(67.7)	91(69.5)	87(65.9)	
Gender				0.093				0.564				0.95
Female	82(43.4)	47(50.0)	35(36.8)		85(43.4)	45(45.9)	40(40.8)		130(49.4)	64(48.9)	66(50.0)	
Male	107(56.6)	47(50.0)	60(63.2)		111(56.6)	53(54.1)	58(59.2)		133(50.6)	67(51.1)	66(50.0)	
Tumor stage				0.01*				0.187				0.09
Stage I-II	80(42.3)	49(52.1)	31(32.6)		76(38.8)	43(43.9)	33(33.7)		123(48.2)	69(53.9)	54(42.5)	
Stage III-IV	109(57.7)	45(47.9)	64(67.4)		120(61.2)	55(56.1)	65(66.3)		132(51.8)	59(46.1)	73(57.5)	
T				0.02*				0.008**				0.05
T1-T2	16(9.0)	13(14.6)	3(3.4)		18(17.8)	14(29.8)	4(7.4)		47(17.9)	30(22.9)	17(12.9)	
T3-T4	161(91.0)	76(85.4)	85(96.6)		83(82.2)	33(70.2)	50(92.6)		216(82.1)	101(77.1)	115(87.1)	
N				0.044*				0.623				0.096
N0	75(43.1)	45(51.1)	30(34.9)		50(49.5)	25(53.2)	25(46.3)		134(51.0)	74(56.5)	60(45.5)	
N1-N2	99(56.9)	43(48.9)	56(65.1)		51(50.5)	22(46.8)	29(53.7)		129(49.0)	57(43.5)	72(54.5)	
M				0.038*				0.014*				0.345
M0	156(88.1)	83(93.3)	73(83.0)		62(61.4)	35(74.5)	27(50.0)		140(80.5)	71(83.5)	69(77.5)	
M1	21(11.9)	6(6.7)	15(17.0)		39(38.6)	12(25.5)	27(50.0)		34(19.5)	14(16.5)	20(22.5)	

Chi-square test

* p<0.05; ** p<0.01.
