# Supplementary figures and images for: A novel strategy for precise prognosis management and treatment option in colon adenocarcinoma with TP53 mutations
Source: Front Surg. 2023 Feb 9;10:1079129. doi: 10.3389/fsurg.2023.1079129 (PMC9947352; doi:10.3389/fsurg.2023.1079129)

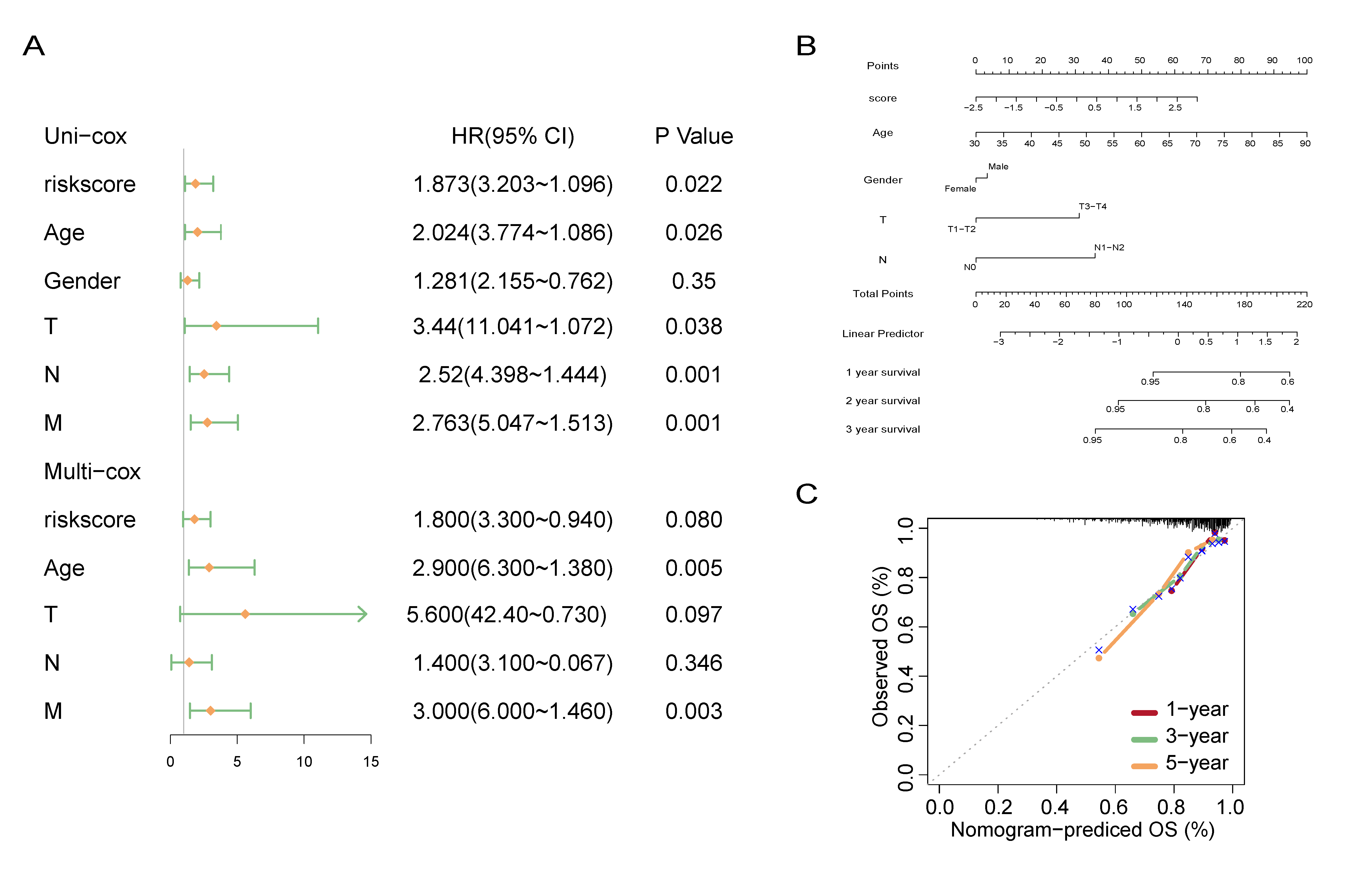

Supplement: Supplementary file 2 [file Image1.tif]
